# Death of Dr. Charles Winne

**Published:** 1877-06

**Authors:** 


### Death of Dr. Charles Winne.

A special meeting of the Erie County Medical Society was held May 11th at the Medical College, to take action on the death of Dr. Charles Winne.

The meeting was called to order by the Vice President, Dr. E. Storck, who stated its object and paid a tribute to the memory of the deceased. The following announcement was then made by Dr. John Boardman :

*Mr. President and Gentlemen:* It becomes my sad duty to announce to you the death of Charles Winne, which took place at 10.30 P. M., Wednesday, May 9, 1877, at his residence in this city.

Charles Winne was born in Albany, N. Y., October, 1811. At fifteen years of age he entered Union College. After he graduated he entered the office of William Bay, M. D., one well known and respected by the old residents of Albany. He was a young man of marked ability, for while yet a student he performed several surgical operations for some of the other physicians. In 1832 he was placed in their cholera hospital, the record of its mortality being much improved under his care. He received his medical degree in New York in 1833, and the same year made his home in this city. The same year commenced those public medical services which occupied so much of his lifetime. For a cholera hospital being established in an old stone building near the lake, which was destroyed not many years since, he devoted much of the summers '33 and '34, to the care of the many sick daily brought in there. The medical care at the Poor-house for a time and a surgical attendance of many years at the Buffalo Hospital of the Sisters of Charity fill out these duties.

A partnership was early formed with the late Josiah Trowbridge which continued till the withdrawal of the senior partner from practice, which was renewed on his resuming his practice. Later, he was a partner, for a time, of Dr. Walter Cary.

In the fall of 1851, while performing a surgical operation at the hospital, Dr. Winne's hand became poisoned. For nine weeks his sufferings were great, and much of that time he seemed like Mahomet's coffin suspended in mid air between life and death. It was months before he was able to resume his professional duties. From that injury, in my opinion, his physical and nervous system never fully recovered. * * * * * * *

It seems needless for me here to speak of his abilities, for we knew him. He was a man of strong will, self-reliant, to which he united a mind trained by long and constant study, quick perception, clear judgment, well read not only in medicine and surgery but he had found time to become so famillar with science, art, literature and politics that he was at home with all of them, and hardly could a subject be broached to which he could not bring clear and well-digested thoughts or opinions.

He was a gentleman of the old school, polite, dignified, rather reserved, hating duplicity or smallness, a warm friend or an open enemy, not proud or vaunting, but courteous in his manners to those he met in consultation; many times leading others in such a gentle way that his advice seemed more the suggestions of their own thoughts—yet if necessary, clear, distinct and prompt in advice.

I have known him not only to admire his judgment and medical skill, but to esteem him as a friend and adviser, and can render but a poor tribute to the man I have thought of so highly.

Dr. Boardman closed by moving that a committee of three be appointed to draft suitable resolutions.

Dr. White in seconding the motion spoke of his life and character in feeling and fitting terms.

The motion of Dr. Boardman was carried, and the chair appointed as the committee of three, Dr. John Boardman, Dr. H. N. Loomis, and Dr. John Hauenstein.

Dr. P. H. Strong paid a feeling tribute to the memory of the deceased, and so did Dr. J. S. Trowbridge. The committee on resolutions then reported as follows:

WHEREAS, Death has removed from our midst Charles Winne, the senior member of our Society,, one valued as a physician, counselor and friend.

*Resolved*, That in his death we deeply regret the loss of one whose industry, energy and skilled judgment have made him a piller of strength to all those that leaned upon him.

*Resolved*, That we tender our sincerest sympathies to his family in this their dark hour.

*Resolved*, That the secretary of the Society be directed to transmit a copy of these resolutions to the family of the deceased.

The resolutious were unaminously adopted. Remarks, eulogistic of Dr. Winne, were made by Dr. H. R. Hopkins, Dr. C. C. F. Gay, Dr. J. D. Hill, and Dr. John Hauenstein. The meeting then adjourned.

At half-past four o'clock yesterday afternoon, the funeral services were held at the family residence, conducted by the Rev. Dr. Van Bokkelen, Rector of Trinity Church, and the Rev. Dr. Ingersoll of Niagara Falls. The attendance was large, including many members of the profession.

The remains were last evening taken to Albany for interment.

